# A possible case of Erdheim–Chester Disease

**Published:** 2018

**Authors:** Georgeta Martiniuc, Dana Mihaela Turliuc, Mihaela Miron, C Martiniuc

**Affiliations:** * Suceava County Emergency Hospital, Suceava, Romania; ** ”Professor Doctor Nicolae Oblu” Emergency Hospital Iasi, Iasi, Romania; *** University Hospital in Château-Gontier, France

**Keywords:** proptosis, infiltrative lesions, visual acuity, Erdheim-Chester disease

## Abstract

**Aim:** To present the case of a 57-year-old male with progressive bilateral proptosis.

**Material and method:** The patient presented with bilateral proptosis and strength deficiency on the upper limbs. During hospitalization, the progression was unfavorable; proptosis progressed, causing a severe loss of vision in the left eye (from 0.8 Snellen to NLP).

**Results:** The imagistic investigation revealed bilateral infiltration of the orbits, infiltrative lesions to the mediastinum and the abdomen. The patient was referred to neurosurgery for further management. Surgical orbital decompression was performed with biopsy. The histopathological examination revealed non-Hodgkin small cell lymphoma.

**Conclusions:** Assembling the clinical and paraclinical data we have suspected the possible diagnosis of Erdheim-Chester disease, however, positive diagnosis has not been achieved.

## Introduction

The Erdheim-Chester Disease (ECD) is a rare form of non Langerhans cell histiocytosis. Individuals affected by this disease are typically adults in their 5th–7th decades of life. Males and females are almost equally affected. The ECD has no known etiology, is a multisystemic disease mainly involving the bones, lungs, skin, retro-orbital tissues, central nervous system (CNS), large vessel, kidneys, retroperitoneum and heart [**1**,**2**].

Typical features for ECD are osteosclerosis in femurs and tibia, perinephritic stranding (“hairy kidney”), periaortic infiltration (“coated aorta”) [**2**].

Neurological symptoms represent a prominent feature of ECD and occur in approximately 25%-50% of the patients at the onset or during the course of the disease. Exophthalmos, gaze disturbances, diabetes insipidus, cerebellar syndromes, seizure, and focal mass lesions-related radiculopathy are the recurrent manifestations affecting the CNS, whose lesions are directly responsible for one third of all deaths and have been specifically identified as independent predictive factors of poor prognosis [**2**,**3**].

Orbital infiltration occurs in 25% of the patients and can present as exophthalmos, oculomotor palsies. Mass effect of the retro-orbital lesions might result in the thickening and tortuosity of the optic nerves. The lacrimal glands and orbital muscles, as well as the retro-orbital adipose tissue may be involved with the lesions [**2**,**4**].

Diagnostic criteria: histopathological (foamy histiocytes positive for CD 68 and Negative for CD1a on immunohistochemical staining) and radiological criteria (bilateral, symmetric osteosclerotic lesions) [**2**,**5**,**6**].

## Case report

We reported the case of a 57-year-old male with painful proptosis (approximately 3 weeks) associated with defective ocular motility, decrease of visual acuity and strength deficiency on the upper limbs.

**Ophthalmological examination**

• visual acuity RE (right eye) = 0.9, LE (left eye) = 0.8; 

• OU: proptosis and limitation of adduction, abduction, depression and elevation;

• LE: palpebral ptosis;

• OU: normal pupillary reflex.

**Neurological examination**

No signs of meningeal irritation, brachial diparesis with inability to raise the right arm over 90 degrees; motor shortage for prehension and fist extension, more emphasized on the globally diminished right side reflexes. 

**Electromyography**

Does not detect significant damage to the peripheral nerves of upper limbs.

Initiation of corticotherapy with Medrol 80 mg/ daily (**presumptive diagnosis of myositis**), the patient’s progression is unfavorable with the increase in proptosis and the marked decrease of visual acuity LE (NLP).

**Craniocerebral MRI**

**Fig. 1:**
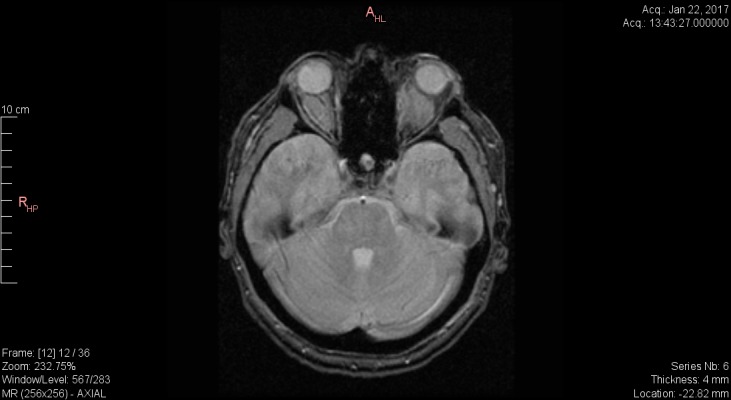
Craniocerebral IRM Axial section T2 fr FSE – infiltrative lesions in retrobulbar space without delimitation from EOM in LE

Retrobulbar space replacing processes; with thickened wall structure, external contours well delimited to the extraocular muscles RE; maximum diameter 25/12/18 mm; without delimitation from medial rectus (MR), superior rectus (SR) and levator palpebrae superioris LE; maximum diameter 35/25/27mm (**Fig. 1**).

**Thoracoabdominal CT**

**Fig. 2:**
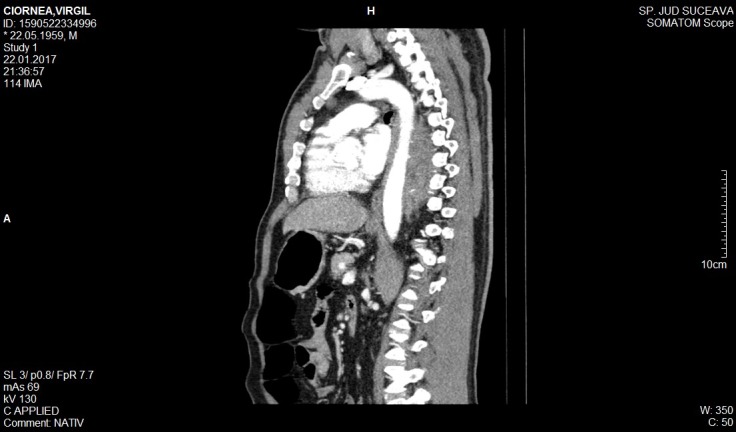
Thoracoabdominal CT - “coated aorta”

**Fig. 3:**
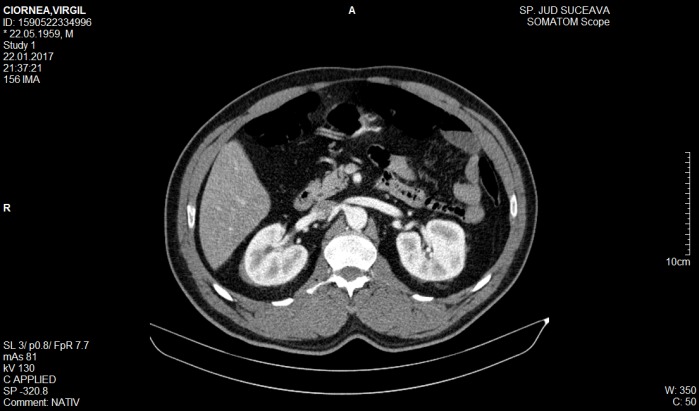
Abdominal CT - “hairy kidney”

Infiltrative tissue lesion in the posterior median cervix, with a maximum thickness of 5.3 cm in the sagittal plane, which incorporates the descending thoracic aorta without deviating it (**Fig. 2**-**3**). The set of lesions described may be compatible with multisystemic infiltrative lymphoma.

It was decided that the patient should be urgently transferred to a university neurosurgical clinic for therapeutic management. Emergency surgical treatment was performed: bilateral orbital decompression by the orbital ceiling frontal resection, microscopic ablation of the left intraorbital formation and partial abortion of the right intraorbital formation.

**Postoperative cranial CT**

**Fig. 4:**
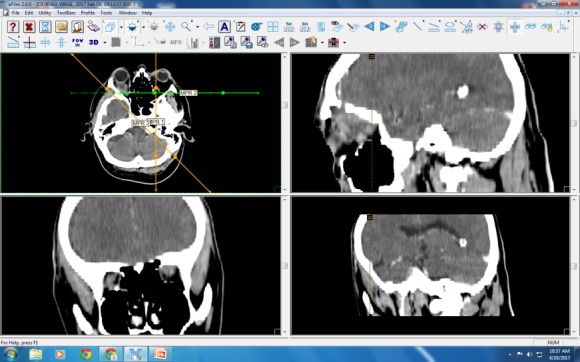
Craniocerebral CT – intraorbital lesions with frontal sinus invasion

**Histopathological examination**

Tumor proliferation consisting of diffuse atypical lymphocytes, intratumoral capillary vessels are present without obvious alterations. Final anatomopathological diagnosis: Small cell diffuse non-Hodgkin’s lymphoma.

**Differential Diagnosis**

• Wegener lymphogranulomatosis (excluded by immunological dosages of pANCA and cANCA antibodies);

• Langerhans/ non Langerhans cells (the diagnosis of certainty is performed by specific immunohistochemical stains from 2 tissue samples).

**Evolution**

The patient is currently under the supervision of Suceava Oncology Clinic, tetraplegic, blind (progressive decrease of visual acuity RE) under antalgic treatment (opiates).

## Discussion

• The rapid unfavorable progression associated with multisystemic manifestation and atypical histopathology suggested a particular form of Langerhans non-Langerhans cells, Erdheim-Chester disease. 

• Corticotherapy changed the appearance of histiocytes by inhibiting the development. 

• Histopathological examination was also sent to Hôpital Pitié-Salpêtrière, Paris, France (insufficient tissue for diagnosis). 

## Conclusions

• Due to the small number of cases reported in literature, of which only 25% presented orbital manifestations, the symptoms could easily be included in the clinical picture of other diseases [**1**-**3**].

• For the diagnosis of certainty, specific immunohistochemical examinations (CD 68 +, CD 163 +, Positive Factor XIIIa, Negative CD1a, Negative CD207) should be performed [**2**,**4**].
